# Prenatal diagnosis – where do we go from here?

**DOI:** 10.1515/medgen-2026-2010

**Published:** 2026-04-16

**Authors:** Christine Fauth, Sarah Verheyen

**Affiliations:** Institute of Human Genetics Medical University of Innsbruck 6020 Innsbruck Austria; Diagnostic and Research Institute of Human Genetics Medical University of Graz Stiftingtalstr. 6 8010 Graz Austria

For many years, prenatal diagnosis primarily focused on the detection of common trisomies in women with advanced maternal age using basic ultrasound and conventional karyotyping of amniotic fluid or chorionic villus samples. During the last two decades, however, prenatal diagnosis has undergone a major transformation which was driven by advances in prenatal imaging and genomic sequencing technologies. Technical improvements of prenatal ultrasound and magnetic resonance imaging (MRI) allow for a detailed fetal assessment during various developmental stages. In parallel, new genetic technologies, particularly chromosomal microarray analysis, exome and genome sequencing have been rapidly integrated into clinical prenatal diagnosis. Consequently, an increasing number of congenital malformations and genetic disorders are now diagnosed during pregnancy.

The articles in this issue of *Medizinische Genetik* address distinct aspects in prenatal diagnosis including the current practice and indications for prenatal testing, the capabilities of modern MRI imaging technologies, the role of fetal pathology in postmortem phenotyping, discordant organ malformations in monozygotic twins, and challenges associated with placental mosaicism.

The article by Sarah Jauch and Philipp Klaritsch provides an overview of current prenatal diagnostic practice and illustrates potential implications of prenatal genetic testing. The authors point out that prenatal diagnosis may influence obstetric care and provide examples of intrauterine therapies for specific genetic disorders.

Not all countries have uniform national standards for prenatal genetic testing. Indications for chromosomal microarray analysis, exome and genome sequencing may differ substantially and are often shaped by societal attitudes and the structure and capacity of the healthcare systems to bear the costs of advanced diagnostic testing. Sarah Verheyen and colleagues review current recommendations for invasive prenatal testing and the selection of an appropriate testing strategy depending on the fetal phenotype.

Two articles in this issue focus on fetal phenotyping. Gregor Kasprian and colleagues provide a radiologist’s perspective on deep fetal phenotyping by modern MRI techniques. They describe main indications for fetal MRI and illustrate that MRI is complementary and for selected questions superior to prenatal ultrasound. In cases with apparently “isolated” brain anomalies like “agenesis of corpus callosum” or “ventriculomegaly” on prenatal ultrasound, sophisticated MRI techniques can reveal subtle additional features, which may point to an underlying genetic disorder and refine the prognosis for neurodevelopmental outcome.

Susanne Sprung and colleagues address the current role of fetal autopsy and describe an interdisciplinary approach of pathology and human genetics. The authors provide selected examples of common and rare disorders and illustrate the role of autopsy in comprehensive characterization of fetal phenotypes, the evaluation of stillbirths, the assessment of placental pathology, and in quality control of prenatal ultrasound findings after termination of pregnancy.

The article of Heidrun Schuligoi and colleagues discusses potential genetic, epigenetic and environmental causes in monochorionic twins with discordant malformations.

Patricia Döttelmeyer and Christine Fauth focus on chromosomal mosaicism detected in chorionic villus samples. The authors provide a review of the current literature including risk estimates of true fetal involvement, laboratory work-up, implications for genetic counselling, diagnostic pitfalls and effects upon placental function. They point out that knowledge of placental mosaicism is essential for the interpretation of non-invasive prenatal testing (NIPT) results which are based on cell free placenta DNA in maternal blood.

While technological achievements in imaging and genetics now allow for diagnosis of a growing number of disorders during pregnancy, the rapid shift of these technologies into routine prenatal diagnosis also poses new challenges and raises specific ethical issues [1–3].

The main motivation of parents for prenatal screening by ultrasound and/or NIPT is to be reassured about the health status of their developing fetus. In case of an abnormal finding, parents are offered invasive genetic testing in order to obtain clarity. A clear diagnosis is the basis for detailed information of the parents, obstetric management, potential treatment options and parental decision-making about the continuation or termination of pregnancy.

However, the results of prenatal tests are not always clear and simple, and interpretation of data (in particular of genomic sequencing data) critically depends on the clinical phenotype (which may evolve over time) and the current state of knowledge. Parents are often not prepared to obtain uncertain results and may unexpectedly find themselves in a vicious circle of anxiety, emotional distress, and the pressure of timely decision-making in the context of legal regulations on pregnancy termination [1, 3–5].

To minimize uncertainty, many professional guidelines on prenatal genomic sequencing and chromosomal microarray analysis recommend against returning variants of unknown significance to parents. However, even in cases with a clear genetic diagnosis the variability of the clinical phenotype often hinders accurate prediction. The same applies to genetic variants with reduced penetrance (e. g. certain recurrent microdeletions and microduplications), mosaic findings, or (ultra-)rare disorders with limited clinical information [3, 4].

To ensure that parents benefit from prenatal diagnosis, there are some essential requirements [1, 2, 4–7]:

*Thorough pre-test counselling*
  In order to obtain valid consent, parents must be informed about the purpose, potential outcomes, and implications of prenatal testing. They should be aware of uncertainties related to prenatal testing and the currently limited knowledge.
  The decision for or against prenatal diagnosis is a personal deliberate process which has to respect personal, ethical and religious beliefs.
*Experience in communicating prenatal testing results and consistency of information*
  In case of an abnormal test result, the information on the expected clinical phenotype of the unborn child should be balanced, accurate and include potential treatment options as well as information on long-term care and financial and psychosocial support for families with a handicapped child. The information given by health care providers from different disciplines should be consistent, requiring close interdisciplinary communication. A major challenge is to communicate uncertain findings to expectant parents and to guide them through this stressful phase.
*Sufficient time for decision making in cases with abnormal results*
  A well-considered decision requires time. However, the indication for exome or genome sequencing is often based on fetal anomalies not detected until the 20-week fetal anomaly scan or even later. When test results are available (after 10–14 days), the fetus may already be viable. These situations are extremely stressful for parents and doctors and often accompanied by worries about timing, as a potential decision to terminate a pregnancy at the threshold of fetal viability would require a feticide.

Uncertain results of prenatal testing are expected to diminish over time as more information on genetic variants and prenatal phenotypes becomes available. In recent years, major efforts have been undertaken to enhance systematic comprehensive fetal phenotyping [8]. Hopefully, this will solve some of the problems related to uncertain prenatal testing results.

However, other challenges will remain. In this editorial we can only touch a few of the complex ethical issues related to prenatal testing which affect parents and clinicians in different ways and also involve legal and fundamental socio-ethical aspects.

Critical questions in laboratory and clinical genetics are: Which findings should be reported to parents (and which are not to be reported)? Should parents have a right to obtain any available genetic information on their future child including information on late-onset disorders?

For clinicians involved in prenatal diagnosis, litigation worries present a substantial burden which may lead to increased prenatal testing. Health care providers fear potential legal implications, if they don’t offer comprehensive prenatal testing and the child later turns out to be affected by a genetic disorder that could have been diagnosed prenatally [5].

A general concern in bioethical debates related to prenatal testing and in particular screening (like NIPT) is selection against disability, often referred to as “liberal eugenics”. Extensive prenatal screening may result in discrimination of disabled persons and make them feel less welcome in our society.

Prenatal diagnosis operates within a multifaceted tension field that requires balancing between parental autonomy, physical and mental health of the pregnant women, interests of the unborn child, medical, legal, ethical, religious, and psychosocial considerations. It is our responsibility to integrate all these aspects which requires a close interdisciplinary dialogue. Things are complex and there will be no simple answers.
